# Mind Perception of Robots Varies With Their Economic Versus Social Function

**DOI:** 10.3389/fpsyg.2018.01230

**Published:** 2018-07-18

**Authors:** Xijing Wang, Eva G. Krumhuber

**Affiliations:** Department of Experimental Psychology, University College London, London, United Kingdom

**Keywords:** robots, economic function, social function, mind perception, emotion, cognition, moral treatment

## Abstract

While robots were traditionally built to achieve economic efficiency and financial profits, their roles are likely to change in the future with the aim to provide social support and companionship. In this research, we examined whether the robot’s proposed function (social vs. economic) impacts judgments of mind and moral treatment. Studies 1a and 1b demonstrated that robots with social function were perceived to possess greater ability for emotional experience, but not cognition, compared to those with economic function and whose function was not mentioned explicitly. Study 2 replicated this finding and further showed that low economic value reduced ascriptions of cognitive capacity, whereas high social value resulted in increased emotion perception. In Study 3, robots with high social value were more likely to be afforded protection from harm, and such effect was related to levels of ascribed emotional experience. Together, the findings demonstrate a dissociation between function type (social vs. economic) and ascribed mind (emotion vs. cognition). In addition, the two types of functions exert asymmetric influences on the moral treatment of robots. Theoretical and practical implications for the field of social psychology and human-computer interaction are discussed.

## Introduction

With the rapid advancement of technology, robots are becoming increasingly intelligent and autonomous entities with the ability to fulfill a variety of tasks. Traditionally, such tasks have been restricted to the industrial setting in which robots were used for factory work, such as car manufacturing, building construction, and food production (Bekey, 2012; Kim and Kim, 2013). Thus, robots were built mainly to achieve economic efficiency and financial profits for the corporate world (i.e., economic value). This conception, driven mainly by instrumental considerations, is likely to change as robots are starting to penetrate the social sphere (Matarić, 2006; Dautenhahn, 2007; Lin et al., 2011). With new application areas ranging from healthcare to education and domestic assistance (Gates, 2007; Friedman, 2011; Bekey, 2012; Dahl and Boulos, 2013), the places that robots are going to occupy in the future alongside humans are likely to be social in nature. As such, robots will become integral parts in our daily lives, appearing in public and private domains. Facing this new era raises significant questions about the prospective relation between man and machine. Would the value type of the robots influence the extent to which we perceive life-like qualities in them and consider them as worthy of care and moral concern? The current research addresses this issue by exploring the impact of the robot’s proposed function (social vs. economic) on mind attribution and moral treatment.

### Anthropomorphism

The question whether robots belong to the category of living or non-living entities has received considerable attention from researchers in different fields (Gaudiello et al., 2015). Anthropomorphism, i.e., the tendency to attribute humanlike characters to nonhuman entities, is a widely observed phenomenon in human–robot interaction (Epley et al., 2007; Airenti, 2015). Despite ongoing debates about the underlying functions and mechanisms of anthropomorphism (see Duffy, 2003; Levillain and Zibetti, 2017), attributing life-like qualities to robots whose behavior follow preprogrammed scripts is an intuitive process (Zawieska et al., 2012). This is because mind attribution does not involve a rational analysis by stepping inside others’ heads, but is instead shaped by how targets appear to us and how we feel towards them (Coeckelbergh, 2009). People automatically respond to and often overestimate the social cues emitted by the robot’s humanlike face, voice, and/or body movements (Reeves and Nass, 1996; Nass and Moon, 2000). We then impose the same source evaluation criteria (e.g., social stereotypes and heuristics) to these robots in the same fashion as done with human targets (e.g., Nass and Lee, 2001). For example, a smiling robot activates the memory exemplar of a nice person which in turn creates the impression of a sociable robot (Kiesler and Goetz, 2002; Powers and Kiesler, 2006).

When it comes to the way in which people perceive and treat robots, the predictive power of anthropomorphism is robust. As such, people are more likely to cooperate with robots (Powers and Kiesler, 2006), engage, and have enjoyable interactions with them (Walker et al., 1994; Koda and Maes, 1996), when they are anthropomorphic in appearance. Humans also tend to show increased empathy by trying to save them from possibilities of suffering (Riek et al., 2009). Despite these important findings, anthropomorphism has been largely criticized on its lack of conceptual and methodological consensus (Zawieska et al., 2012). In particular, anthropomorphism has been usually treated as a unidimensional concept, including multiple components with variations across studies, such as intellect, sociability, mental abilities, cognitions, intentions, and emotions (e.g., Bartneck et al., 2008; Nowak et al., 2009).

### Cognition and Emotion

Full humanness or humanlike characteristics entail both *higher order cognition* (e.g., rationality and logic) and *emotion* (e.g., feelings and experience, Kahneman, 2011; Waytz and Norton, 2014). These two core capacities, i.e., cognition and emotion, also map onto the two dimensions of mind perception (Gray et al., 2007), the conceptualization of humanness (Haslam, 2006; Haslam et al., 2008), as well as the two primary dimensions of social perception (Fiske et al., 2007). Specifically, higher order cognition corresponds to agency, human uniqueness traits, and competence, while emotion is related to experience, human nature, and warmth (Waytz and Norton, 2014). Interestingly, it has been recently demonstrated that people automatically evaluate a target’s mind along these two dimensions (e.g., Gray et al., 2007), and such target can either be living entities, such as humans and animals (Fiske et al., 2007; Gray et al., 2007), or non-living entities, such as corporate brands (Aaker et al., 2010) and robots (Waytz and Norton, 2014). Furthermore, research suggests that emotion is perceived to be more essential to humanness than cognition (e.g., Willis and Todorov, 2006; Ybarra et al., 2008; Gray and Wegner, 2012) and it also plays a significant role in how we treat others (Gray and Wegner, 2009).

Robots have long been perceived as non-human or a subhuman species, designed as a tool to serve our needs but not to deserve our respect and equal concern as our fellow human beings (Friedman et al., 2003; Dator, 2007). This is mainly because they are attributed with only higher order cognition but not feelings and emotions (e.g., Haslam, 2006; Gray et al., 2012; Kim and Kim, 2013). In other words, it is the ascribed emotion and experience, but not cognition and agency, make a target deserve moral concerns (Gray and Wegner, 2009). This could mainly due to the fact that robots were historically designed out of purely pragmatic considerations in terms of the economic functions they support, i.e., efficiency and automation (Young et al., 2009; Chandler and Schwarz, 2010). Although this might result in attributions of higher order cognition (i.e., artificial intelligence), robots in the traditional sense are not seen as sentient beings (Gray and Wegner, 2012; Kim and Kim, 2013). Hence, people associate robots more with cognition- than emotion-oriented jobs (Loughnan and Haslam, 2007) and express more comfort in outsourcing jobs when those require cognition (Waytz and Norton, 2014).

Over the last 15 years, a number of social robots have been developed with the aim to provide social support and companionship. These include, for example, Nursebot Pearl and Care-o-bot 3 for medical and social care (Pollack et al., 2002; Graf et al., 2009), as well as robots such as Nao or Pepper (Robotics, 2010) for companionship. Unlike traditional robots, social robots are designed to socially engage humans (DiSalvo et al., 2002; Duffy, 2003; Walters et al., 2008), being specifically built to form an attachment (Darling, 2014). Given that this task emphasizes companionship and affective sharing, traits which are closely associated with feelings and emotions (e.g., Fiske, 1992; Gray et al., 2007; Cuddy et al., 2008), it is possible that the robot’s social value increases perceptions of its capacity for emotional experience. Knowledge about the proposed function of a robot could therefore trigger different attributions of mind.

Theoretical support for this assumption comes from social role theory. People infer the traits of a target from his/her socially defined categories (e.g., woman, mother, and manager), or the perceived social role s/he performs (Hoffman and Hurst, 1990; Wood and Eagly, 2012). For instance, women are perceived as affectionate and warm, while men are judged as agentic and competent given their typically ascribed gender role (i.e., caregiver vs. breadwinner, Spence et al., 1979; Eagly and Wood, 2011). Such influence of predefined categorical roles goes beyond the classic domain of gender and applies not only to human targets but also to non-living entities. Of direct relevance to the current study, previous research has demonstrated that businesspeople are typically seen to have higher capacity for cognition but to lack emotions in comparison to housewives and artists (Fiske et al., 2002; Loughnan and Haslam, 2007). Similarly, non-profit organizations dedicated to furthering social support are ascribed higher levels of emotion, but a lower degree of cognition compared to companies which aim for economic growth (Aaker et al., 2010). In line with this thinking, perceptions of robots’ emotional and cognitive ability could vary with their social and economic function, respectively. Moreover, these different values could have downstream consequences for moral consideration, in the sense that social/emotional skills afford rights as a moral patient, making robots deserve protection from harm (Waytz et al., 2010; Gray et al., 2012).

## The Present Research

With robots being built for the purpose to add value to humans’ lives (Kaplan, 2005), such value can differ depending on whether it is to fulfill social or economic needs. In particular, in this paper, we define social value/function as the tendency to provide social support and companionship for human society. In contrast, we refer to economic value/function as the tendency to make financial profits and benefits for the corporate world. The primary aim of this paper is to examine whether the proposed function of robots exerts a significant influence on mind perception. In addition, we aimed to test whether such function also shapes subsequent moral treatment.

While previous research has largely focused on the appearance of robots and revealed the benefits of human resemblance (e.g., Hegel et al., 2008; Krach et al., 2008; Powers and Kiesler, 2006; Riek et al., 2009), the very reason for robots’ creation is in fact to assist humans with their built-in functions (e.g., Zhao, 2006). While a social function in the service of affiliation may imply the capacity for feelings and emotion, an economic function could indicate cognitive skills such as thinking and planning. To test this hypothesis, we conducted four studies in which we systematically varied information about the robot’s function using text-based descriptions, while controlled other information such as visual appearance.

Studies 1a and 1b were designed to examine whether the distinct function of robots differentially affects the two dimensions of mind. Given that the ability to experience and express emotions is essential for social connection, we predicted that robots with social value would attract higher ratings of emotion, but not cognition, than those with economic value. By contrast, the perceived mind of robot with economic function and control group would not differ given that the stereotypical function of robot is economic in nature (Gates, 2007; Friedman, 2011).

Study 2 aimed to replicate the findings observed in the first studies and further test whether there exists a dissociation in people’s beliefs between the function of a robot and its type of mind. For this, we made robots either lack or gain value by assigning high vs. low economic and social functionality. Given that robots have historically been associated with tasks that require cognitive rather than social skills (Lohse et al., 2007; Kim and Kim, 2013; Darling, 2014), we hypothesized that losing economic value would lead to reduced perceptions of cognition. By contrast, gaining social value should result in increased emotion attribution.

Finally, Study 3 was to replicate the finding of Study 2 and further test the moral consequences caused by its value type. As such, gaining social value, rather than economic value, should increase perceived emotional ability/experience of the robot. In addition, people should be most willing to protect robots with (high) social value from harm, given that perceived emotion/experience is tied to moral concern (Gray and Wegner, 2009).

### Sensitivity Analysis

Given our primary aim, calculations were based on the effect of value type (social vs. economic) on mind perception. According to sensitivity power analysis (G^∗^Power, Faul et al., 2007), minimal effect sizes of *f* = .13 (Study 1A, *N* = 151, value type: between-subjects, mind dimension: within-subjects), *f* = .15 (Study 1B, *N* = 50, value type and mind dimension: within-subjects), *f* = .08 (Study 2, *N* = 172, value level and mind dimension: within-subjects, value type: between-subjects), and *f* = .11 (Study 3, *N* = 110, value level, value type, and mind dimension: within-subjects) could be detected under standard criteria (*α* = 0.05 two-tailed, *β* = 0.80), respectively.

### Study 1

In Studies 1a and 1b, we sought to provide initial evidence for the hypothesis that mind attribution varies with the robot’s function. To this end, robots were described as having either social or economic value. In order to test whether people assign by default economic rather than social value to robots, we also included a control condition in which the robot’s function was not mentioned explicitly. We predicted that robots with social value would be ascribed higher levels of emotion, but not cognition, compared to those in the economic and control condition. Given that the stereotypical function of robots is to serve economic purposes (e.g., Gates, 2007; Friedman, 2011), we expected that robots with economic function and those whose functions are not mentioned explicitly (i.e., control condition) would be perceived similarly.

### Study 1A

#### Method

##### Participants

One hundred and fifty-one participants (85 women, *M*_age_ = 24.8, *SD* = 6.98, 86% Caucasian, 5% Asian, and 9% others) were recruited from public areas in central London (i.e., public libraries and cafés) and took part in the study on a voluntary basis. The experimental design included the value type (economic vs. social vs. control) as a between-subject variable. Participants were randomly assigned to one of three conditions, resulting in approximately fifty people in each group. The study was conducted with ethical approval from the Department of Experimental Psychology at University College London, United Kingdom.

#### Material and Procedure

Short text-based descriptions of four robots were created which served as profile information: e.g., “*This robot can quickly learn various movements from demonstrators and also make additional changes either to optimize the behavior or adjust to situations.”* In order to assign economic versus social value, these profile descriptions were combined with information about the robot’s corresponding function: (a) economic condition: e.g., “*Therefore, this robot can work as a salesperson in stores and supermarkets, guiding customers to different products and answering their inquiries”* and (b) social condition: e.g., “*Therefore, this robot can work as a social caregiver, keeping those socially isolated/lonely people accompanied, reminding them of their daily activities and having conversations with them*.*”* In the control condition, no further information about the robot’s function was provided besides its basic profile description (please see Appendix A).

Pilot-testing with a separate group of participants (*N* = 56) confirmed that robots with social function were indeed perceived to be higher in their social value^1^ (*M* = 59.5, *SD* = 14.4 vs. *M* = 29.3, *SD* = 21.8) and lower in their economic value (*M* = 67.3, *SD* = 12.9 *M* = 78.9, *SD* = 13.6) compared to those with economic function, *p*s ≤ 0.002.

The study was completed on paper. After providing informed consent, participants were presented with text-based descriptions of four robots (of the same value type within a condition) which they rated on two dimensions of mind: (a) emotion (the capacity to *experience emotions*, *have feelings*, and *be emotional*, *α*s ≥ 0.93) and (b) cognition (the capacity to *exercise self-control*, *think analytically*, and *be rational*, *α*s ≥ 0.93)^2^. To distract participants from the aims of the study, they also evaluated each target on several filler items, i.e., how good-looking, durable, and lethargic the robot appears to be. All responses were made on 100-point scales ranging from 0 (*not at all*) to 100 (*very much*). The order of presentation of the four robot descriptions was counterbalanced across participants.

#### Results and Discussion

Mean values for emotion and cognition were computed by combining the three items of each measure into a single score and averaging across the four robot descriptions (emotion: *α =* 0.94, cognition: *α =* 0.93) within each dimension. A repeated measures ANOVA with mind dimension (emotion vs. cognition) as a within-subjects factor and value type (social vs. economic vs. control) as a between-subjects factor was performed.

A significant interaction between mind dimension and value type emerged, *F*(2,148) = 4.44, *p* = 0.013, $\eta_{p}^{2}$ = 0.057. Pairwise comparisons revealed that the ascribed ability for emotional experience was significantly higher for robots with social than those with economic value (*M*_difference_ = 12,4, *p* = 0.001) and those in the control group (*M*_difference_ = 14.3, *p* < 0.001), with no significant difference between the latter two conditions, *p* = 0.918 (see **Table 1**). In contrast, perceived cognition did not differ as a function of value type, *p*s ≥ 0.792.

**Table 1:** Means and standard deviations (in brackets) of all measures in Study 1.

	Emotion	Cognition
**Study 1A**		
Social	25.9 (21.7)	68.3 (26.3)
Economic	13.5 (13.3)	68.5 (20.5)
Control	11.6 (12.1)	64.6 (21.5)
**Study 1B**		
Social	27.3 (26.3)	57.2 (27.3)
Economic	17.1 (22.3)	58.0 (31.0)
Control	18.8 (24.7)	56.0 (28.4)


*Higher scores correspond to higher levels of perception on that dimension.*

As predicted, knowledge about a robot’s proposed function critically moderated judgments of mind. Specifically, assigning social value to robots increased levels of perceived emotion. By contrast, attributions of cognition did not change in robots with economic function and those whose value was not mentioned explicitly, suggesting that robots are predominantly considered to possess economic rather than social function.

### Study 1B

Study 1b aimed to replicate the findings of the first study with a within-subjects design. In addition, we wanted to describe robot’s social and economic value more directly. That is, instead of letting participants infer a robot’s value from its proposed function, we provided explicit information in the sense that the robots are created to provide social support and companionship (i.e., social value) versus financial benefits and corporate profits (i.e., economic value), respectively.

#### Method

##### Participants

Fifty participants (30 women, *M*_age_ = 27.4, *SD* = 6.86, 84% Caucasian, 6% Black, and 10% others) were recruited from public areas in central London (i.e., public libraries and cafés) and took part in the study on a voluntary basis. The experimental design included the value type (economic vs. social vs. control) as within-subject variable. The study was conducted with ethical approval from the Department of Experimental Psychology at University College London, United Kingdom.

#### Material and Procedure

The same text-based descriptions as in Study 1a served as profile information about the robots. In order to assign economic versus social value, these profile descriptions were combined with information about the robot’s corresponding function: “*According to a recent business (social) analysis, this robot is predicted to be of profound economic (social) value. By economic (social) value, we mean the expected financial benefits and corporate profits (social support and companionship) they are going to bring to the corporate world (human society).*” In the control condition, no further information about the robot’s function was provided besides its basic profile description.

The study was completed on paper. After providing informed consent, participants were presented with text-based descriptions of the three types of robots (economic, social, and control) which they had to rate on two dimensions of mind: (a) emotion (the capacity to *experience emotions*, *have feelings*, and *be emotional*, *α*s ≥ 0.89) and (b) cognition (the capacity to *exercise self-control*, *think analytically*, and *be rational*, *α*s ≥ 0.82). To distract participants from the aims of the study, they also evaluated each target on several filler items which were identical to those used in the previous study. All responses were made on 100-point scales ranging from 0 (*not at all*) to 100 (*very much*). To avoid any carryover effects, the robot in the control condition was always presented first. The order of the two experimental conditions (economic vs. social) was counterbalanced across participants.

#### Results and Discussion

Mean values for emotion and cognition were computed by combining the three items of each measure into a single score. A repeated measures ANOVA with value type (economic vs. social vs. control) and mind dimension (emotion vs. cognition) as within-subjects factors was performed. Replicating the finding of Study 1A, a significant interaction between value type and mind dimension was obtained, *F*(2,98) = 3.82, *p* = 0.025, $\eta_{p}^{2}$ = 0.072. Pairwise comparisons showed that ratings of emotion were significantly higher for robots with social value than economic value (*M*_difference_ = 10.2, *p* = 0.010) and those in the control group (*M*_difference_ = 8.5, *p* = 0.014, see **Table 1**), with no significant difference between the latter two conditions, *p* = 0.628. In contrast, the perceived cognition did not differ significantly among the three value types, *p ≥* 0.456. This pattern of results replicates what was found in the first study. Specifically, social value positively influenced a robot’s perceived capacity for emotional experience, but not higher order cognition.

### Study 2

The aim of Study 2 was to replicate the finding of Study 1 devoid of any information about the robots’ basic function (e.g., ability to learn various movements). To this end, we used visual stimuli, which were kept constant across conditions, to serve as the basic profile information. In addition, we tested for a dissociation in people’s beliefs between the function of a robot and its type of mind. For this, robots were made to either lack or gain value by assigning high vs. low economic and social functionality. If the two types of functions project distinct values (i.e., efficiency and productivity vs. caring and communality), attributions of cognition and emotion should be affected differently. Based on the finding that robots are primarily considered to be of economic value (i.e., high economic value), but not of social value (i.e., low social value), we hypothesized that losing economic value (i.e., low vs. control) would result in reduced levels of ascribed cognition. In contrast, gaining social value (i.e., high vs. control) should lead to increased emotion perception.

#### Method

##### Participants

One hundred and seventy-two participants (78 women, *M*_age_ = 23.9, *SD* = 6.26, 79% Caucasian, 6% Asian, and 15% others) were recruited online through a participant testing platform. All participants were fluent English speakers and took part in the study on a voluntary basis. The two-factor experimental design included the value type (economic vs. social) as a between-subject variable, and the value level (high vs. low vs. control) as a within-subject variable. Participants were randomly assigned to one of the two value type conditions, resulting in eighty-six people in each group. The study was conducted with ethical approval from the Department of Experimental Psychology at University College London, United Kingdom.

#### Material and Procedure

Color images of three robots, displaying neutral expressions with direct gaze, were chosen from publicly accessible Internet sources. Images were edited and cropped using Adobe Photoshop so that only the head and shoulder of the robots remained visible. In addition, we centered the head and created a uniform white background. Images measured 300 × 300 pixels.

To assign economic versus social value, text-based descriptions of the robot’s corresponding function were created which also systematically differed in the value level. In the economic condition, it was stated that “*According to a recent business report, this robot is predicted to be of high (low) economic value. By economic value, we mean the expected financial benefits and corporate profits they are going to bring to the corporate world.*” In the social condition, it was stated that “*According to a recent social report, this robot is predicted to be of high (low) social value. By social value, we mean the expected the social support and companionship they are going to bring to the human society.*” In the control condition, no textual description of the robot was provided. The matching between robot image and robot type was counterbalanced across participants.

The study was conducted online using Qualtrics software (Provo, UT, United States). After providing informed consent, participants were presented with images together with text-based descriptions of three robots that differed in their value level (high, low, and control) within the same value type. The matching between visuals and text was counterbalanced, so that each robot image was equally likely to appear with each value level description across participants. For each stimulus target, participant provided explicit evaluations on two dimensions of mind: (a) emotion (the capacity to *experience emotions*, *have feelings*, and *be emotional*, *α*s ≥ 0.89) and (b) cognition (the capacity to *exercise self-control*, *think analytically*, and *be rational*, *α*s ≥ 0.85). As in the previous studies, filler items were included to distract participants from the aims of the study. All responses were made on 100-point scales ranging from 0 (*not at all*) to 100 (*very much*). To avoid any carryover effects, the robot in the control condition was always presented first. The order of the two experimental conditions (high value, low value) was randomized.

#### Results and Discussion

Mean values for emotion and cognition were computed by combining the three items of each measure into a single score. A repeated measures ANOVA with value level (high vs. low vs. control) and mind dimension (emotion vs. cognition) as within-subjects factors, and value type (economic vs. social) as a between-subjects factor was conducted.

Importantly, a significant three-way interaction between value level, vale type, and mind dimension emerged, *F*(2,340) = 4.81, *p* = 0.009, $\eta_{p}^{2}$ = 0.028. Specifically, for ratings of emotion, the interaction between value type and value level was significant, *F*(1.91,325) = 5.19, *p* = 0.007, $\eta_{p}^{2}$ = 0.030. Further pairwise comparisons showed that the ascribed ability for emotional experience was greater for robots with high social value than low social value (*M*_difference_ = 10.0, *p* < 0.001, see **Table 2**) and those in the control group (*M*_difference_ = 12.2, *p* < 0.001), with no significant difference between the latter two conditions, *p* = 0.313. Perceptions of emotion were unaffected by the level of a robot’s economic value, *p*s*≥* 0.342.

**Table 2:** Means and standard deviations (in brackets) of all measures in Study 2.

	Emotion	Cognition
**Social**		
High	36.9 (27.9)	60.0 (25.6)
Control	24.7 (22.5)	57.2 (29.4)
Low	26.9 (25.1)	60.1 (26.3)
**Economic**		
High	24.1 (23.2)	59.1 (28.5)
Control	21.9 (21.8)	60.1 (28.4)
Low	23.0 (22.6)	51.3 (27.6)


*Higher scores correspond to higher levels of perception on that dimension.*

For ratings of cognition, the interaction between value type and value level was also significant, *F*(1.97,335) = 4.13, *p* = 0.017, $\eta_{p}^{2}$ = 0.024, but the observed patterns were different from those of emotion. Specially, attributions of cognitive ability decreased for robots with low economic value than high economic value (*M*_difference_ = -7.8, *p* = 0.005, see **Table 2**) and those in the control group (*M*_difference_ = -8.8, *p* = 0.004) with no significant difference between the latter two conditions, *p* = 0.740. Perceptions of cognition were unaffected by the level of a robot’s social value, *p*s*≥* 0.313.

Replicating the findings of Studies 1a and 1b, robots with (high) social value were also ascribed greater emotional ability than those with (high) economic value (*M*_difference_ = 12.8, *p* = 0.001, see **Table 2**). No such difference in robot functionality was observed for attributions of cognitive ability, *p* = 0.829.

In sum, the social value of robots significantly increased emotion judgments which is consistent with the previous findings. In addition, there was a dissociation between the perceived function of a robot and its type of mind. While losing economic value diminished ratings of cognition, gaining social value increased perceptions of emotional experience.

### Study 3

Study 3 aimed to replicate the finding of Study 2 that gaining social value increases perceived emotional experience. In addition, it aimed to explore the moral consequences in terms of whether intentions to harm differ with the type (economic vs. social) and level (high vs. low) of a robot’s value. If perceptions of experience are tied to the attribution of moral rights, robots with high social value should be more likely to afford protection from harm (Gray et al., 2012).

#### Method

##### Participants

One hundred and ten participants (35 women, *M*_age_ = 23.5, *SD* = 6.31, 82% Caucasian, 8% Asian, and 10% others) were recruited online through a participant testing platform. All participants were fluent English speakers and took part in the study on a voluntary basis. The two-factor experimental design included the value type (economic vs. social) and the value level (high vs. low) as within-subject variables. The study was conducted with ethical approval from the Department of Experimental Psychology at University College London, United Kingdom.

#### Material and Procedure

The same four text-based descriptions as in Study 2 were used to assign high vs. low economic and social value to robots.

The study was conducted online using Qualtrics software (Provo, UT, United States). After providing informed consent, participants were instructed to imagine a hypothetical scenario in which an electromagnetic wave was going to be generated in the lab. They were told that they could save three out of the four robots from the undesirable electromagnetic shock by placing them into a shielded room. The four robots systematically differed in their value level (high, low) and value type (economic, social) as described in the vignettes.

To measure (1) the intention to *harm*, participant’s task was to indicate for each robot category *how willing they were to sacrifice the robot to receive a shock* in order to spare the other three. To measure (2) the perception of *emotion*, they further indicated *how much pain* and *how sad the robot would feel* if it were to receive a shock (*α*s ≥ 0.92). All responses were made on seven-point scales ranging from 0 (*not at all*) to 7 (*very much*). The presentation order of the four robot descriptions was randomized.

#### Results and Discussion

Repeated measures MANOVA with value level (high vs. low) and value type (economic vs. social) as within-subjects factors were performed on measures of emotion and harm. Firstly, the main interaction between value type and value level was highly significant, *F*(2,108) = 22.3, *p* < 0.001, $\eta_{p}^{2}$ = 0.292.

Regarding ratings of emotion, a significant interaction between value type and value level was obtained, *F*(1,109) = 35.7, *p* < 0.001, $\eta_{p}^{2}$ = 0.247. Replicating the findings of Study 1 and 2, perceived ability for emotional experience was elevated for robots with high social value (*M*_difference_ = 1.19, *M*_difference_ = 1.33, *M*_difference_ = 1.27, **Table 3**) compared to the other three robot types, *t*s(109) ≥ 7.01, *p*s ≤ 0.001, *d*s ≥ 1.34, for which ratings did not significantly differ from each other, /*t*s(109)/ ≤ 1.06, *p*s ≥ 0.293.

**Table 3:** Means and standard deviations (in brackets) of all measures in study 3.

	Emotion	Intention to harm
**Social**		
High	3.62 (2.28)	2.95 (1.86)
Low	2.43 (1.68)	5.56 (1.56)
**Economic**		
High	2.29 (1.66)	3.60 (2.00)
Low	2.35 (1.71)	5.45 (1.63)


*Higher scores correspond to higher levels of perception/behavioral tendency on that dimension.*

Regarding intentions to harm, a main effect of value level for willingness to harm, *F*(1,109) = 166, *p* < 0.001, $\eta_{p}^{2}$ = 0.604, showed that people were less likely to sacrifice robots for an electric shock when they were of high value (*M* = 3.27) than low value (*M* = 5.51). In addition, the interaction between value type and value level was significant, *F*(1,109) = 6.97, *p* = 0.010, $\eta_{p}^{2}$ = 0.060. Paired-sample *t*-tests further revealed that participants’ intention to harm was the lowest for robots with high social value (*M*_difference_ = 0.65, *M*_difference_ = 2.50, *M*_difference_ = 2.61, see **Table 3**) in comparison to the other three robot types, *t*s(109) ≥ 2.87, *p*s ≤ 0.005, *d*s ≥ 0.55.

Correlational analysis confirmed a significant association between emotion ratings and intentions to harm, *r*(440) = -0.331, *p* < 0.001. As such, the higher the level of ascribed emotional experience, the less the inclination to endorse harm, which is consistent with findings by Gray and Wegner (2009). High social value therefore afforded rights as a moral patient, making robots capable of emotional experience as well as eligible to deserve protection from harm.

## General Discussion

Ever since their creation, robots have been considered as a non-human species, being developed to fulfill users’ goals without the need to deserve equal moral concern (e.g., Kim and Kim, 2013). This could derive from the fact that they were granted humanlike cognition, but not emotional capacity (e.g., Gray and Wegner, 2009, 2012). The current research aimed to explore whether the proposed function of robots, i.e., economic vs. social, shapes ascriptions of mind and in particular emotional experience. In addition, we examined the downstream consequences of robots’ functionality for moral treatment.

Studies 1a and 1b demonstrated that robots with social function received higher ratings of emotion, but not cognition, compared to those with economic function and whose function was not mentioned explicitly. Study 2 replicated the findings of the first two studies by showing that robots with (high) social function were assigned greater levels of emotion than those with (high) economic function. In addition, we demonstrated that losing economic value led to lower ascriptions of cognitive capacity while gaining social value resulted in increased emotion perception. Study 3 finally revealed that robots with high social value are likely to be afforded protection from harm by gaining rights as a moral patient. Such moral concern was significantly associated with their levels of ascribed emotional experience.

Together, the findings have important theoretical and practical implications. First, consistent with prior findings, the current study also demonstrates that mind perception is an intuitive and subjective process (e.g., Epley et al., 2007; Wang and Krumhuber, 2017b), and is a two-dimensional phenomenon, consisting of emotion/experience and higher order cognition/agency (Gray et al., 2007; Złotowski et al., 2014). Crucially, such perception is subject to the proposed function of a robot (social vs. economic). While previous studies on human-robot interaction have focused on the importance of humanlike form and behavior (e.g., Powers and Kiesler, 2006; Hegel et al., 2008; Krach et al., 2008; Riek et al., 2009), the present research demonstrates that distinct functions can elicit different inferences of mental ability.

Furthermore, there exists a dissociation between the robot’s function and its type of mind. While an economic function implies cognitive skills, it is the social function that makes robots capable of experiencing emotions in the eyes of the observer. This evidence is consistent with prior studies on human targets (Fiske et al., 2002; Loughnan and Haslam, 2007; Aaker et al., 2010), showing that levels of ascribed cognition/competence and emotion/warmth vary within the same entity. Thus, people seem to apply similar heuristics in response to humans and robots (e.g., Nass and Lee, 2001).

Due to the traditional role of robots in industrial settings, the existing gap in mind perception between humans and robots can be largely explained by the perceived lack of emotions and fundamental experiences (e.g., Haslam, 2006; Gray and Wegner, 2012). For example, Eyssel et al. (2010) showed that anthropomorphic inferences of robots increased with their ability to express emotions. Similarly, Złotowski et al. (2014) found that perceived emotionality, but not intelligence, contributed to the ascription of human traits in robots. Equipping robots with emotional skills, thereby highlighting their social value, could make them more humanlike and evoke empathic responses. Given that moral concern is associated with perceived experience, but not agency (Gray et al., 2012), it is the social value which confers moral rights and patiency as shown in the present research. This evidence is particularly timely in the context of recent discussions about the legal protection of robots (Darling, 2014). Although robots will never be fully sentient as defined by biological principles, the moral standing of those possessing social value will likely to be different from those fulfilling economic needs (Coeckelbergh, 2009).

Despite the importance of these findings, it is worth pointing out some limitations of the current research which could serve as avenues for future studies. Although robots’ social value acts as a critical moderator in people’s willingness to ascribe emotions, such perceived emotion is still much lower compared to the level of ascribed cognition. Assigning social value to robots therefore seems to increase their level of perceived emotional capacities only to a limited extent. This could due to the fact that we only employed minimal manipulations in the current study, i.e., simple textual description of robots’ function type. Future studies in human–computer interaction could let participants directly interact with robots that have been built to fulfill economic and social needs, respectively.

Furthermore, it would be interesting to examine the behavioral responses to robots of diverse functionality. Beliefs about a target’s mind have been shown to affect various outcomes, including likeability, social attention, joint-task performance, and emotional attachment (e.g., de Melo et al., 2013; Wykowska et al., 2014; Martini et al., 2016). With the rapid rise of social robots in society, future studies might be aimed at testing whether the emotions ascribed to robots based on their social value significantly influence people’s tendency to trust and cooperate. While first responses could result in feelings of uneasiness and discomfort (e.g., Gray and Wegner, 2012; Waytz and Norton, 2014; Wang and Krumhuber, 2017a) due to infrequent exposure to robots of this kind (Kätsyri et al., 2015), the social nature of robots may act as the basis for human bonding and attachment at a later stage (Darling, 2014; Dryer, 1999).

## Ethics Statement

All subjects gave informed consent in accordance with the Declaration of Helsinki. The protocol was approved by the Department of Experimental Psychology at University College London, United Kingdom.

## Author Contributions

XW and EK conceived the presented idea. XW carried out the experiments and wrote the manuscript with support from EK.

## Conflict of Interest Statement

The authors declare that the research was conducted in the absence of any commercial or financial relationships that could be construed as a potential conflict of interest.

## Footnotes

1 Further principle component analysis (PCA) showed that four robots with assigned social value loaded on the same factor while the robots with assigned economic value loaded on the other, explaining 65.6% of the variance in combination, KMO = 0.68, Bartlett’s test of sphericity, χ^2^(28) = 196, *p* < 0.001.

2 Further principle component analysis (PCA) showed that the three items measuring emotion loaded on the same factor while the three items measuring cognition loaded on the other, explaining 84.5% of the variance in combination, KMO = 0.76, Bartlett’s test of sphericity, χ^2^(15) = 3507, *p* < 0.001.
