# Supplementary material for: Mind Perception of Robots Varies With Their Economic Versus Social Function
Source: Front Psychol. 2018 Jul 18;9:1230. doi: 10.3389/fpsyg.2018.01230 (PMC6058296; doi:10.3389/fpsyg.2018.01230)
Supplement: Supplementary file 1 [file Data_Sheet_1.docx]

**Appendix A**

**Text-based descriptions of four robots in Study 1A**

Robot 1

*Profile information*: This robot can recognize human faces and voices, understand human voice command and carry on conversations.

*Economic function/economic condition:* Therefore, this robot can work as a salesperson in stores and supermarkets, guiding customers to different products and answering their inquiries.

*Social function/social condition:* Therefore, this robot can work as a social care giver keeping those socially isolated/lonely people accompanied, reminding them of their daily activities and having conversations with them.

*Control condition*: none

Robot 2

*Profile information*: This robot can carry, move around and place things at precise locations. This robot can also move to different locations as commanded.

*Economic function/economic condition*: Therefore, this robot can provide door-to-door courier/delivery service, including getting goods from different shelves in storage, packing products, and then sending them to different places for customers.

*Social function/social condition*: Therefore, this robot can provide healthcare assistance for those people who have lost autonomy, including lifting them from bed to toilet, helping them with bathing and showering, and taking them to different places that they desire.

Control condition: none

Robot 3

*Profile information*: This robot can quickly learn varied movements from demonstrators and also make additional changes either to optimize the behavior or adjust to situations.

*Economic function/economic condition:* Therefore, this robot can work as a factory worker, performing various tasks, such as operating and optimizing production line equipment, grading and feeding raw materials at an optimal rate, and monitoring the production process.

*Social function/social condition:*Therefore, this robot can work as a babysitter and housekeeper, doing various household chores, such as cooking dinners that meet the needs of the family, changing diapers and feeding babies according to his/her needs, and playing with the baby and other family members.

Control condition: none

Robot 4

*Profile information*: This robot can systematically learn your email/ message writing style and response pattern and make everything look like you've just written an email/message yourself.

*Economic function/economic condition:*Therefore, this robot can help people write to customers, colleagues and even their boss (of course the user choose to click on the ‘send’ button or not), and thus can save people lots of time and largely increase work efficiency.

*Social function/social condition:*Therefore, this robot can help people write to friends, family members and even their partner (of course the user choose to click on the ‘send’ button or not), and thus can keep people’s social, family, and love life more alive.

Control condition: none
